# Epidemiology and infection control of carbapenem resistant *Acinetobacter baumannii* and *Klebsiella pneumoniae* at a German university hospital: a retrospective study of 5 years (2015–2019)

**DOI:** 10.1186/s12879-021-06900-3

**Published:** 2021-11-27

**Authors:** Patrick Chhatwal, Ella Ebadi, Frank Schwab, Stefan Ziesing, Ralf-Peter Vonberg, Nicolas Simon, Svetlana Gerbel, Dirk Schlüter, Franz-Christoph Bange, Claas Baier

**Affiliations:** 1 Institute for Medical Microbiology and Hospital Epidemiology, Hannover Medical School, Carl-Neuberg-Straße 1, 30625 Hannover, Germany; 2 Institute of Hygiene and Environmental Medicine, Charité, University Medicine Berlin, Hindenburgdamm 27, 12203 Berlin, Germany; 3 Centre for Information Management (ZIMt), Hannover Medical School, Carl-Neuberg-Straße 1, 30625 Hannover, Germany

**Keywords:** *Acinetobacter baumannii*, *Klebsiella pneumoniae*, Carbapenem resistant, Infection control, Epidemiology, Screening

## Abstract

**Background:**

Carbapenem resistant (CR) *Klebsiella pneumoniae* (Kp) and *Acinetobacter baumannii* (Ab) are emerging multidrug resistant bacteria with very limited treatment options in case of infection. Both are well-known causes of nosocomial infections and outbreaks in healthcare facilities.

**Methods:**

A retrospective study was conducted to investigate the epidemiology of inpatients with CR Kp and CR Ab in a 1500-bed German university hospital from 2015 to 2019. We present our infection control concept including a weekly microbiologic screening for patients who shared the ward with a CR Kp or CR Ab index patient.

**Results:**

Within 5 years, 141 CR Kp and 60 CR Ab cases were hospitalized corresponding to 118 unique patients (74 patients with CR Kp, 39 patients with CR Ab and 5 patients with both CR Ab and CR Kp). The mean incidence was 0.045 (CR Kp) and 0.019 (CR Ab) per 100 inpatient cases, respectively. Nosocomial acquisition occurred in 53 cases (37.6%) of the CR Kp group and in 12 cases (20.0%) of the CR Ab group. Clinical infection occurred in 24 cases (17.0%) of the CR Kp group and in 21 cases (35.0%) of the CR Ab group. 14 cases (9.9%) of the CR Kp group and 29 cases (48.3%) of the CR Ab group had a history of a hospital stay abroad within 12 months prior to admission to our hospital. The weekly microbiologic screening revealed 4 CR Kp cases caused by nosocomial transmission that would have been missed without repetitive screening.

**Conclusions:**

CR Kp and CR Ab cases occurred infrequently. A history of a hospital stay abroad, particularly in the CR Ab group, warrants pre-emptive infection control measures. The weekly microbiologic screening needs further evaluation in terms of its efficiency.

**Supplementary Information:**

The online version contains supplementary material available at 10.1186/s12879-021-06900-3.

## Background

Carbapenem resistant (CR) *Klebsiella pneumoniae* (Kp) and *Acinetobacter baumannii* (Ab) are emerging multidrug resistant bacteria [1, 2]. The World Health Organization lists them both as critical pathogens that pose a relevant threat to human health [3]. Both species are known to cause nosocomial infections such as pneumonia or bloodstream infection [4]. Infections caused by CR Kp or CR Ab can lead to increased mortality, in particular when initial antibiotic treatment is inadequate [5, 6]. In-hospital costs are also significantly increased in patients with CR Kp and CR Ab [7, 8].

The frequency of carbapenem resistance among Kp und Ab isolates varies significantly between countries and is comparatively low in Germany [9, 10]. The frequency of CR Kp seems to increase slightly in Germany [11], whereas there is a decreasing trend for carbapenem resistance in Ab isolates [12].

Spread of CR Kp in Europe is mainly driven by nosocomial transmission [13]. CR Kp and CR Ab can cause nosocomial outbreaks in different patient care settings [14, 15]. There are guidelines for healthcare facilities addressing infection control of CR Gram-negative bacteria (GNB) [16–19]. These infection control measures include isolation in single rooms, contact precautions and targeted microbiologic screening for patients at increased risk of CR GNB colonization.

In this study, we present data on the epidemiology of inpatients with CR Kp and CR Ab in a 5 year period at a university hospital in Germany. Moreover, we discuss our infection control concept which included a microbiologic screening aiming at detection of potential nosocomial transmission of CR Kp and CR Ab.

## Methods

### Setting, study design, data acquisition and definitions

We conducted a retrospective study at Hannover Medical School, a university hospital in northern Germany with about 1500 beds. All methods were performed in accordance with the relevant guidelines and regulations. The in-house infection control management software and the microbiology laboratory information system were used to identify all inpatients with CR Kp or CR Ab from January 2015 to December 2019*.* The corresponding inpatient stays were determined. Each inpatient stay was considered a separate CR Kp or CR Ab case. A stay of an inpatient who harbored CR Kp and CR Ab at the same time was counted as a case for each group. Demographic (e.g., age, gender) and clinical data (e.g., length of stay) were collected from the hospital’s Enterprise Clinical Research Data Warehouse and medical charts.

An infection was defined as follows: (i) CR Kp or CR Ab was identified in a microbiologic specimen taken for diagnostic purposes, and (ii) the patient showed signs and symptoms of infection, and (iii) the infection was documented in the medical chart.

In the absence of a history of CR Kp or CR Ab a nosocomial acquisition was defined as a positive microbiologic sample obtained after the second day of the hospital stay.

The microbiology laboratory information system was used to identify screening specimens.

### Statistical analysis

The incidence of CR Kp and CR Ab was calculated as the number of CR Kp and CR Ab inpatient cases per 100 inpatient cases.

In a univariate descriptive analysis, epidemiologic and clinical characteristics of CR Kp and CR Ab patients were compared and differences were tested using Chi-square test for categorical variables, and Wilcoxon rank sum test for continuous variables. Number and percentages were calculated for categorical variables, while the median and the interquartile range were used for continuous variables. Moreover, a multivariate analysis comparing both groups was performed using logistic regression with the outcome “CR Ab”. The multivariate analysis was performed stepwise forward with the significance level of 0.05 for including and 0.06 for the stay of a parameter in the model (all parameters except bone marrow transplantation and urinary tract infection were considered). Calculations were done in SPSS (IBM SPSS Statistics for Windows, Version 25.0. Armonk, NY, USA, IBM Corp.).

### Microbiologic diagnostic

The microbiologic diagnostic was performed at the Institute for Medical Microbiology and Hospital Epidemiology at Hannover Medical School. The microbiologic laboratory is accredited according to ISO 15189. Screening samples targeting CR GNB (including CR Kp and CR Ab) were plated on a selective agar. During the study period we used at first an in-house produced Mac Conkey agar supplemented with ertapenem (1 mg/L) and since the end of 2018 the CHROMagar™ mSuperCARBA™ (CHROMagar, Paris, France). Species were identified with a matrix-assisted laser desorption/ionization time of flight mass spectrometry system (bioMérieux, Marcy-l’Étoile, France).

Antibiotic susceptibility was tested with the VITEK 2 system (bioMérieux, Marcy-l’Étoile, France). The Merlin Micronaut system (Merlin Diagnostika, Bornheim-Hesel, Germany) was used for confirmation and re-testing of increased minimal inhibitory concentrations (MICs). This microdilution-based method was also used for testing of reserve antibiotics such as colistin. For categorization of susceptibility, the respective standards of the European Committee on Antimicrobial Susceptibility Testing (EUCAST) were followed. The 2019 EUCAST changes (e.g., nomenclature of the intermediate category as “susceptible, increased exposure”) were implemented in the microbiologic laboratory after the end of the study period.

Kp isolates with increased MIC for carbapenems (e.g., MIC for ertapenem > 0.5 mg/L) were tested for carbapenemases. In the study period different methods such as disc diffusion (e.g., KPC and MBL Confirm Kit, Rosco Diagnostica, Taastrup, Denmark; if applicable in combination with an in-house modified Hodge test), immunochromatography (RESIST-4 O.K.N.V., Coris BioConcept, Gembloux, Belgium) or polymerase chain reaction (Xpert® Carba-R for carbapenemase producing organisms, Cepheid, Sunnyvale, California, USA) were used. In special cases isolates were also sent to the German National Reference Laboratory for Multidrug Resistant GNB. Ab isolates were not tested for carbapenemases in the routine workflow (as carbapenem resistance in Ab is largely caused by specific carbapenemases, such as OXA-23) [20].

For the analysis of the antimicrobial susceptibility distribution, each patient’s first CR Kp or CR Ab microbiologic sample was considered.

### Infection control management

Kp and Ab isolates that were categorized as intermediate or resistant for meropenem and/or imipenem were classified as CR. Kp isolates carrying a carbapenemase were always classified as CR regardless of the primarily measured carbapenem MIC. Patients with CR Kp or CR Ab were housed in private rooms (single room isolation). Personal protective equipment (gloves, gowns and a surgical mask) for the staff were mandatory when entering the patient’s room. Patient rooms were cleaned with a commercial disinfectant containing phenoxyethanol, N,N-bis-(3-Aminopropyl)dodecylamine and benzalkonium chloride. After discharge of the patient, the cleaning procedure was performed twice. Disposable medical products, stored open in the patient room, got discarded.

Patients with a history of a hospital stay within the last 12 months in a foreign country with a CR GNB prevalence higher than in Germany or with a known history of CR GNB carriage were isolated preemptively in a single room at admission. Those patients got also immediately screened for multidrug resistant GNB (rectal swabs, groin swabs, respiratory specimens from ventilated patients and swabs from wounds if applicable).

During the stay of a CR Kp or CR Ab positive patient (so called index patient), all other patients on the same ward were screened once a week (weekly “point prevalence” screening). It was also recommended to additionally screen all patients on this ward 1 week and 4 weeks after discharge of the index patient. In the case of a CR Kp index patient, rectal swabs of other patients were recommended. In the case of a CR Ab index patient, rectal and groin swabs were recommended. In intensive care units, additional respiratory specimens were taken from ventilated patients for screening purpose for both types of index patients. If a presumptive transmission was detected by this screening (e.g., similar phenotypic resistance pattern) a pulsed-field gel electrophoresis (PFGE) was carried out.

## Results

### Epidemiology and clinical characteristics

**Fig. 1:**
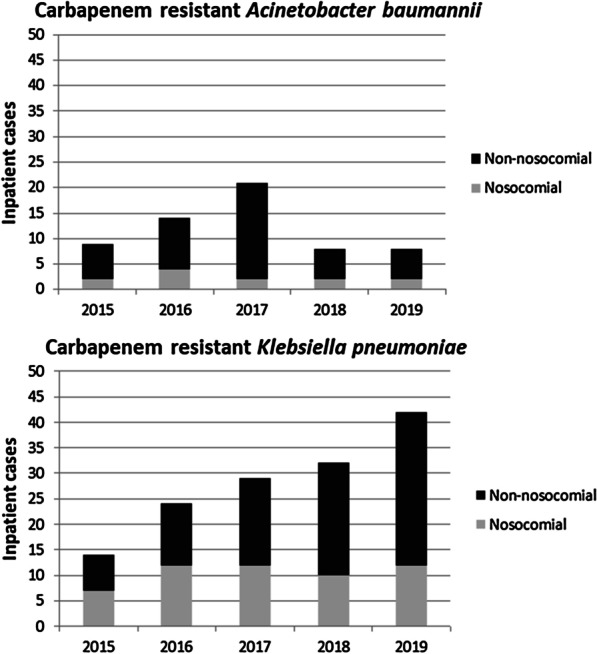
Annual distribution of carbapenem resistant *Acinetobacter baumannii* inpatient cases and carbapenem resistant *Klebsiella pneumoniae* inpatient cases. In the absence of a history of CR Kp or CR Ab a nosocomial acquisition was defined as a positive microbiologic sample obtained after the second day of the hospital stay

In the study period overall 313,464 inpatient cases (distinct hospital stays) corresponding to 229,183 inpatients (i.e., on average 1.4 cases per patient) were recorded at our institution. Among these were 141 CR Kp and 60 CR Ab cases. Figure 1 shows the annual numbers of the CR Kp and CR Ab cases. The average incidence was 0.045 (CR Kp) and 0.019 (CR Ab) per 100 inpatient cases, respectively. The cumulative 201 CR Kp and CR Ab cases corresponded to 118 unique patients (74 patients with CR Kp, i.e., 0.03% of all patients; 39 patients with CR Ab, i.e., 0.02% of all patients; and 5 patients with both CR Ab and CR Kp, i.e., 0.002% of all patients). A total of 38 patients with either CR Kp or CR Ab had more than one inpatient stay in the study period.

Table 1 shows the results of the univariate analysis of selected epidemiologic and clinical parameters of the CR Kp and CR Ab cases. The complete list of parameters is provided in Additional File 1. There were 137 cases (97.2%) in the CR Kp group colonized, predominantly at the rectal site. Twenty-four cases (17.0%) of the CR Kp group had an infection. Colonization was already known prior to the onset of infection in 20 of those cases (14.6% of the patients with CR Kp colonization). Four cases (2.8% of the entire CR Kp group) developed an infection without having a positive colonization sample.

**Table 1:** Epidemiologic and clinical characteristics (selected parameters) of the 201 inpatient cases with carbapenem resistant *Klebsiella pneumoniae* and carbapenem resistant *Acinetobacter baumannii* (univariate analysis)

Parameter	CR *Klebsiella pneumoniae*	CR *Acinetobacter baumannii*	p-value*
Basic epidemiologic and clinical information			
Total number of cases	141 (100%)	60 (100%)	–
Nosocomial cases	53 (37.6%)	12 (20.0%)	**0.015**
Female cases	40 (28.4%)	12 (20.0%)	0.215
Cases with an ICU episode in the stay	54 (38.3%)	29 (48.3%)	0.186
Cases with surgery	73 (51.8%)	30 (50.0%)	0.818
Cases with transplantation	12 (8.5%)	7 (11.7%)	0.484
Median age in years (IQR)	53 (37–64)	53.5 (30.5–65.5)	0.840
Median overall length of stay in days (IQR)	20 (9–45)	24 (13.5–44)	0.427
Median length of isolation precautions in days (IQR)	13 (6–27)	19.5 (8–42.5)	0.098
Previous hospital stays (cases can have a hospital stay in Germany and abroad in the past 12 months)			
Cases with a hospital stay within the past 12 months in Germany	121 (85.8%)	47 (78.3%)	0.190
Cases directly transferred from a German hospital	30 (21.3%)	14 (23.3%)	0.747
Cases with a hospital stay abroad within the past 12 months	14 (9.9%)	29 (48.3%)	**< 0.001**
Cases directly transferred from a hospital abroad	5 (3.5%)	20 (33.3%)	**< 0.001**
ICU stay within the 12 past months	49 (34.8%)	23 (38.3%)	0.628
Distribution of cases according to specialty			
Anesthesia	6 (4.3%)	2 (3.3%)	0.760
Cardiology	4 (2.8%)	1 (1.7%)	0.626
Dermatology	1 (0.7%)	2 (3.3%)	0.160
Gastroenterology	3 (2.1%)	8 (13.3%)	**0.001**
Gynecology	1 (0.7%)	0 (0%)	0.513
Hematology and Oncology	11 (7.8%)	6 (10.0%)	0.608
Heart and thoracic Surgery	38 (27.0%)	6 (10.0%)	**0.008**
Infectious Diseases	1 (0.7%)	1 (1.7%)	0.531
Neurosurgery	2 (1.4%)	6 (10.0%)	**0.004**
Neurology	2 (1.4%)	2 (3.3%)	0.374
Nephrology	4 (2.8%)	1 (1.7%)	0.626
Oral maxillofacial Surgery	0 (0%)	1 (1.7%)	0.124
Pediatric Gastroenterology	4 (2.8%)	0 (0%)	0.188
Pediatric Surgery	4 (2.8%)	0 (0%)	0.188
Pediatric Cardiology	1 (0.7%)	1 (1.7%)	0.531
Pediatric Pulmonology	5 (3.5%)	1 (1.7%)	0474
Plastic Surgery	1 (0.7%)	4 (6.7%)	**0.013**
Pulmonology	1 (0.7%)	2 (3.3%)	0.160
Rheumatology	0 (0%)	2 (3.3%)	**0.029**
Trauma Surgery	24 (17.0%)	12 (20.0%)	0.614
Urology	13 (9.2%)	0 (0%)	**0.015**
Visceral Surgery	15 (10.6%)	2 (3.3%)	0.089
Distribution of cases according to positive samples site (multiple positive body sites possible)			
Bile	1 (0.7%)	0 (0%)	0.513
Blood	7 (5.0%)	3 (5.0%)	0.992
Nasopharyngeal mucosa	14 (9.9%)	20 (33.3%)	**< 0.001**
Rectal	94 (66.7%)	27 (45.0%)	**0.004**
Respiratory tract secretions	18 (12.8%)	17 (28.3%)	**0.008**
Skin	12 (8.5%)	26 (43.3%)	**< 0.001**
Transplant lung perfusion fluid	2 (1.4%)	3 (5.0%)	0.136
Urine	23 (16.3%)	3 (5.0%)	**0.029**
Vascular catheter	3 (2.1%)	4 (6.7%)	0.108
Wound/intraoperative	16 (11.3%)	21 (35.0%)	**< 0.001**
Other sample site	3 (2.1%)	3 (5.0%)	0.274
Colonization and infection (multiple types of infection possible)			
Cases with colonization	137 (97.2%)	55 (91.7%)	0.085
Cases with infection	24 (17.0%)	21 (35.0%)	**0.005**
Bloodstream infection	7 (5.0%)	3 (5.0%)	0.992
Peritonitis	1 (0.7%)	1 (1.7%)	0.531
Pneumonia	5 (3.5%)	6 (10.0%)	0.066
Skin/Soft tissue and surgical site infection	9 (6.4%)	13 (21.7%)	**0.001**
Urinary tract infection	6 (4.3%)	0 (0%)	0.105
Cases with co-colonization			
CR *Escherichia coli*	10 (7.1%)	5 (8.3%)	0.759
CR *Pseudomonas aeruginosa*	13 (9.2%)	7 (11.7%)	0.596
CR “other GNB”	4 (2.8%)	6 (10.0%)	**0.033**
Methicillin-resistant *Staphylococcus aureus*	9 (6.4%)	10 (16.7%)	**0.023**
Vancomycin-resistant *Enterococcus faecium*	44 (31.2%)	18 (30%)	0.866

*CR* carbapenem resistant, *ICU* intensive care unit, *IQR* interquartile range, *Kp* *Klebsiella pneumoniae*, *Ab* *Acinetobacter baumannii,* *GNB* gram-negative bacteria

*2 tailed p-value, Chi-square test for categorical parameters and Wilcoxon rank sum test for continuous parameters. Significant results are displayed in bold

Fifty-five cases (91.7%) of the CR Ab group were colonized. The rectal site, skin and nasopharyngeal mucosa were most common. Twenty-one cases (35.0%) of the CR Ab group had an infection. Colonization was already known prior to the onset of infection in 15 of those cases (27.3% of the patients with CR Ab colonization). Five cases (8.3% of the entire CR Ab group) developed the infection without having a positive colonization sample and in one patient colonization was detected after infection. The results of the multivariate analysis comparing the CR Kp group to the CR Ab group are shown in Table 2. Nearly half of the patients with CR Ab (48.3%) had a history of a hospital stay abroad, which was a significant higher proportion compared to the CR Kp group.

**Table 2:** Multivariate analysis comparing carbapenem resistant *Klebsiella pneumoniae* and carbapenem resistant *Acinetobacter baumannii* (outcome: carbapenem resistant *Acinetobacter baumannii;* logistic regression analysis)

Parameter	OR (CI 95)	p-value
Cases with a hospital stay abroad within the past 12 months	4.853 (1.809–13.018)	0.002
Gastroenterology	23.181 (4.091–131.345)	< 0.001
Positive sample site “rectal”	0.080 (0.027–0.241)	< 0.001
Positive sample site “skin”	14.941 (4.514–49.453)	< 0.001
Positive sample site “wound/intraoperative”	5.034 (1.748–14.496)	0.003
Positive sample site “nasopharyngeal””	6.972 (2.238–21.723)	0.001
Co-Colonization with “CR other GNB”	8.342 (1.534–45.374)	0.014

*OR* odds ratio, *CI 95* 95% confidence interval, *CR* carbapenem resistant, *GNB* gram-negative bacteria

Twenty CR Ab cases were directly transferred from a hospital abroad (Greece: n = 3, Tunisia: n = 3, Poland: n = 2, Africa [not further specified], Bosnia and Herzegovina, Croatia, Egypt, Kuwait, Lebanon, Morocco, Russian Federation, Spain, Thailand, Turkey, Ukraine: all n = 1) and 5 CR Kp cases (Morocco: n = 2, Lebanon: n = 1, Greece: n = 1, Romania: n = 1).

Four patients received a transplant lung which already carried CR Kp and CR Ab (2 lungs with CR Ab, 1 lung with CR Kp and 1 lung with CR Ab and CR Kp) at the time of organ transplantation.

### Antimicrobial susceptibility

There were 123 microbiologic samples (79 Kp and 44 Ab isolates) included in the analysis. The results of the phenotypic antimicrobial susceptibility testing can be found in Additional file 2. Thirty-six Kp isolates (45.6%) had a carbapenemase: 16 isolates with OXA-48, 13 isolates with KPC, 5 isolates with NDM, 1 isolate with both NDM and OXA-48 and 1 isolate with IMI-2. In 12 of the 16 Kp isolates with OXA-48 the meropenem MICs were ≤ 2 mg/L (susceptible). In the other 4 Kp isolates with OXA-48 the meropenem MICs were 8 (intermediate), ≥ 16, > 16 and > 16 mg/L (the latter 3 resistant). The meropenem MICs in the Kp isolates with the other carbapenemases (overall n = 20) were > 8 mg/L (15 isolates > 16 mg/L) and therefore all were meropenem resistant. Five Ab isolates (11.4%) were colistin resistant; all had a MIC > 4 mg/L.

### Weekly prevalence screening

In the study period 6267 inpatients were screened at least once. Overall, 11,137 screening samples were taken (in average 1.8 samples per inpatient). The screening detected 4 patients with CR Kp whose isolates proved identical to the index isolate when compared by PFGE. There were no presumptive Ab transmissions detected by the screening.

In average, 1567 inpatients had to be screened to detect one transmission. The 4 transmission events were caused by 3 independent index patients in different years and different medical departments (2016, 2017 and 2019; visceral surgery, heart and thoracic surgery, hematology and oncology). All 3 index patients carried a CR Kp isolate with a carbapenemase (1 KPC, 2 OXA-48).

**Fig. 2:**
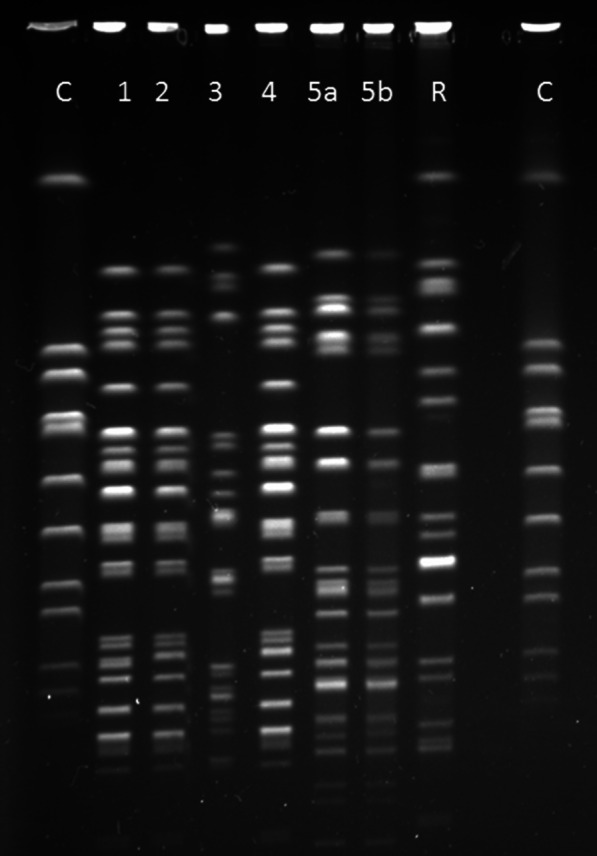
Pulsed-field gel electrophoresis of the carbapenem resistant *Klebsiella pneumoniae* cluster in hematology and oncology. C = Control strain (*Staphylococcus aureus*, National Collection of Type Cultures #8325). R = Reference strain (*Klebsiella pneumoniae*, American Type Culture Collection #700603™). 1: Isolate from index patient. 2 and 4: Isolates highly similar to the index strain from two subsequent cases. 3 and 5a/5b: Unrelated isolates (isolates 5a/5b were recovered from the same patient). PFGE with 1% agarose gel, restriction enzyme Xba I (the original gel is shown in Additional File 3)

In 1 transmission event the index patient and the secondary case shared the same room. In the other 3 transmission events the index patient and the secondary cases were each cared for by the same healthcare staff in neighboring patient rooms. Figure 2 shows the PFGE result of the cluster observed in the hematology and oncology department.

## Discussion

In this study, we analyzed epidemiologic characteristics of patients at a German university hospital that were colonized and/or infected with CR Kp and CR Ab. Moreover, we evaluated the results of a screening program targeting potential transmissions.

The burden of CR GNB including Kp and Ab varies extensively between different countries [21]. For example South-Eastern Europe suffers from a comparatively high healthcare burden of CR Kp and CR Ab [9, 10]. Katchanov et al. found in an 1 year observation period (2015/2016) at another German university hospital 18 patients with CR Ab (0.03% of all patients admitted) and 29 patients with CR Kp (0.05% of all patients admitted) [22]. This finding is quite similar to our results. In contrast, Zarakolu et al. found in a four and a half years study period (2009–2013) 279 of 4105 (6.8%) adult patients to be colonized with CR Kp in Turkey using an active weekly screening approach [23]. Different screening strategies have to be kept in mind when comparing the frequency of CR Kp and CR Ab.

The heterogeneous geographic burden of CR GNB is also reflected to some extent in another finding: Nearly half of the patients with CR Ab in our study had a history of a hospital stay abroad, which was a significant higher proportion compared to the CR Kp group. The introduction of multidrug resistant Ab strains from high prevalence countries has been described for instance in outbreak reports [24]. Additionally, endemicity of carbapenemases (such as OXA-23 and OXA-72) in CR Ab isolates has been found, for example in South-Eastern Europe [25]. The role of a hospital stay abroad as a risk factor for CR Ab is reinforced by the finding, that 7 of the formally 12 CR Ab cases with nosocomial acquisition in our hospital also had a previous hospital stay in a country with high CR GNB burden. It might be possible that these cases had already been colonized with CR Ab on admission, which was not found due to limited screening sensitivity. Limited screening sensitivity is a known issue [26, 27] and might be overcome by repeated screening (for instance later during the hospital stay or after administration of carbapenems).

Nasopharyngeal multidrug resistant Ab colonization is frequently described [27, 28]. We also found frequent nasopharyngeal mucosa colonization (about every third patient) in the CR Ab group—although this was not used as a primary screening site. This observation suggests adding a nasopharyngeal mucosa swab for screening purposes in future. The multivariate analysis showed that nasopharyngeal samples were positively associated with the CR Ab group but rectal samples not. These findings reflect the typical anatomic colonization sites of Enterobacterales and non-fermenting, aerobic GNB.

Nosocomial acquisition was more frequent in the CR Kp group than in the CR Ab group in the univariate analysis. Selective pressure is a well-known risk factor for nosocomial antibiotic resistance development in Kp [29]. The role of antimicrobial stewardship in reducing the burden of multidrug resistant bacteria has been highlighted [30, 31]. Thus, antimicrobial stewardship efforts seem to be necessary in addition to other infection control measures (e.g., screening, isolation, hand hygiene, surface disinfection).

As reported before by others [32], we can confirm that co-colonization with other multidrug resistant bacteria such as Methicillin-resistant *Staphylococcus aureus* and Vancomycin-resistant enterococci occurs frequently. Therefore, potential co-colonization should be kept in mind when practical infection controls measures as cohorting are discussed.

In the study by Katchanov et al. [22] patients with CR GNB were most often cared for by the department of hematology and oncology and visceral surgery. We saw the highest burden in heart and thoracic surgery and trauma surgery for both CR Kp and CR Ab. Colonization and infection with CR Ab and CR Kp have been previously reported in heart and thoracic surgery for instance in patients undergoing heart transplantation [33]. In our cohort, there were 4 heart transplant patients with CR Kp and one of these developed pneumonia with CR Kp. However, the prevalence of CR Kp and CR Ab may differ between different hospitals and medical specialties depending on individual screening strategies among other factors. In the multivariate analysis the gastroenterology department was associated with the CR Ab group compared to the CR Kp group. However, the overall numbers are small here and the 95%-confidence interval is rather large.

The annual distribution of the nosocomial acquired CR Kp and CR Ab cases was quite even during the study period, but we did see an increase of the numbers of non-nosocomial CR Kp cases (Fig. 1). This was mainly due to 4 patients with multiple admissions to the hospital in that time.

An association between colonization and consecutive bloodstream infections has been reported for instance by Kontopoulou et al. [34]. They report that 21.2% of the patients with rectal CR Kp colonization developed bloodstream infections later on. Interestingly, Denkel et al. found an increased risk of infections caused by ESBL producing Enterobacterales among patients who were colonized with ESBL producing Kp compared with ESBL producing *Escherichia coli* [35]. A progression from colonization to infection has also been described for CR Ab (59 infections in 168 colonized patients, i.e., 35.1%) [36]. We also observe the progression from colonization to infection in our cohort, which was more prominent in the CR Ab group. Skin colonization and wound/intraoperative infections were independently associated with the CR Ab group in comparison to the CR Kp group.

Carbapenemase expression is a frequent reason for carbapenem resistance in Kp. The German National Reference Laboratory for Multidrug Resistant GNB reported that in 2018 51.4% of 1531 Kp isolates presenting with phenotypic carbapenem non-susceptibility carried a carbapenemase [37]. That is in line with our findings for Kp (45.6%). In our Kp cohort and for the Enterobacterales analyzed by the German National Reference Laboratory for Multidrug Resistant GNB OXA-48 was the most prevalent type of carbapenemase [37].

Infections caused by CR Ab and CR Kp are difficult to treat. Only few treatment options remain such as colistin [38, 39]. However, colistin resistance is emerging [1, 40]. Fortunately, the majority of the isolates in our cohort were colistin susceptible.

Screening on admission for patients at increased risk of CR GNB colonization is often recommended [16, 17]. Monitoring potential transmission of CR Kp or CR Ab while an index patient with CR Kp or CR Ab is on a ward is of particular interest, as this goes beyond routine procedures. To address this issue, we performed a weekly microbiologic screening of all patients who shared a ward with an index patient. We focused on these two species as both are most problematic in terms of nosocomial transmission in our experience. Noteworthy, within 5 years we did not find a single transmission of CR Ab, but we did find 4 CR Kp transmissions instead. The screening is additional workload for healthcare workers on the wards, increases costs for diagnostic in the microbiological laboratory and sampling may be inconvenient for patients. However, we were able to foster infection control measures in each of the detected transmission events and spread was then interrupted immediately. In addition, screening itself increases awareness and may thereby contribute to good adherence to other prevention measures such as hand hygiene. Nonetheless, we are aware that the number of patients needed to screen was high. Noteworthy, all transmission events in the study period were caused by Kp isolates with a carbapenemase. Thus, one option to increase efficiency might be to perform the prevalence screening merely when a Kp isolate carries a carbapenemase. Moreover, our finding supports that searching for carbapenemases in phenotypically suspicious Enterobacterales is useful in terms of infection control. At the moment, we continue this microbiologic screening regime to re-evaluate its efficiency in the future.

The study has several limitations. First, it is a single center study from a low prevalence country for CR GNB. Therefore, our results may not be transferable to settings in high prevalence areas. Second, the data was collected retrospectively so one has to rely on historical documentation without the possibility of timely interventions. Third, some of the infection control measures used here are resource intensive and might not be feasible in other settings, e.g., extensive microbiologic screening. Fourth, our analysis focuses on epidemiology and infection control. A detailed risk factor analysis for the onset of infection or antibiotic treatment regimens were not within the scope of this study.

## Conclusions

CR Kp and CR Ab occurred infrequently in the study period. Nonetheless, the high infection rate, especially among the CR Ab cases, is noteworthy. A history of a hospital stay abroad, particularly in the CR Ab group, warrants pre-emptive infection control measures such as isolation and screening for CR GNB at admission. The weekly prevalence screening only found 4 transmissions in 5 years, but contributed to rapid interruption of spread. We will further evaluate this procedure in terms of economic and infection control efficiency.

## Supplementary Information

**Additional file 1. **Epidemiologic and clinical characteristics of the 201 inpatient cases with carbapenem resistant *Klebsiella pneumoniae* and carbapenem resistant *Acinetobacter baumannii*.

**Additional file 2. **Results of the phenotypic antimicrobial susceptibility testing.

**Additional file 3. **Pulsed-field gel electrophoresis of the carbapenem resistant *Klebsiella pneumoniae* cluster in hematology and oncology (original gel).

## Data Availability

The datasets generated during and/or analyzed during the current study are available from the corresponding author on reasonable request. Patient data used in this study is confidential according to the German data privacy act, the ethics committee and the data protection commissioner of the Hannover Medical School. To protect patient confidentiality and participant’s privacy, data used for this study can be obtained in anonymous form only according to the data privacy act.

## Abbreviations

CR: Carbapenem resistant
Kp: *Klebsiella pneumoniae*
Ab: *Acinetobacter baumannii*
MIC: Minimal inhibitory concentration
GNB: Gram-negative bacteria
PFGE: Pulsed-field gel electrophoresis
ICU: Intensive care unit
IQR: Interquartile range
OR: Odds ratio
CI95: 95% Confidence interval
