# Supplementary material for: Epidemiology and infection control of carbapenem resistant Acinetobacter baumannii and Klebsiella pneumoniae at a German university hospital: a retrospective study of 5 years (2015–2019)
Source: BMC Infect Dis. 2021 Nov 27;21:1196. doi: 10.1186/s12879-021-06900-3 (PMC8627082; doi:10.1186/s12879-021-06900-3)
Supplement: Supplementary file 1 — Additional file 1. Epidemiologic and clinical characteristics of the 201 inpatient cases with carbapenem resistant Klebsiella pneumoniae and carbapenem resistant Acinetobacter baumannii. [file 12879_2021_6900_MOESM1_ESM.docx]

**Additional file 1**

Epidemiologic and clinical characteristics of the 201 inpatient cases with carbapenem resistant *Klebsiella pneumoniae* and carbapenem resistant *Acinetobacter baumannii*.

| **Parameter** | **CR *Klebsiella pneumoniae*** | **CR *Acinetobacter baumannii*** |
| --- | --- | --- |
| **Basic epidemiologic and clinical information** | | |
| Total number of cases | 141 (100%) | 60 (100%) |
| Nosocomial cases | 53 (37.6%) | 12 (20.0%) |
| Female cases | 40 (28.4%) | 12 (20.0%) |
| Cases with an ICU episode in the stay | 54 (38.3%) | 29 (48.3%) |
| Cases with surgery | 73 (51.8%) | 30 (50.0%) |
| Cases with transplantation | 12 (8.5%) | 7 (11.7%) |
| Cases with heart transplantation | 4 (2.8%) | 0 (0%) |
| Cases with lung transplantation | 4 (2.8%) | 4 (6.7%) |
| Cases with liver transplantation | 4 (2.8%) | 1 (1.7%) |
| Cases with bone marrow transplantation | 0 (0%) | 2 (3.3%) |
| Cases with regular hospital discharge | 86 (61.0%) | 29 (48.3%) |
| Cases discharged by transfer to another healthcare facility | 38 (27.0%) | 20 (33.3%) |
| Cases with discharge reason death | 17 (12.1%) | 11 (18.3%) |
| Median age in years (IQR) | 53 (37-64) | 53.5 (30.5-65.5) |
| Median overall length of stay in days (IQR) | 20 (9-45) | 24 (13.5-44) |
| Median length of isolation precautions in days (IQR) | 13 (6-27) | 19.5 (8-42.5) |
| **Previous hospital stays** (cases can have a hospital stay in Germany and abroad in the past 12 months) | | |
| Cases with a hospital stay within the past 12 months in Germany | 121 (85.8%) | 47 (78.3%) |
| Cases directly transferred from a German hospital | 30 (21.3%) | 14 (23.3%) |
| Cases with a hospital stay abroad within the past 12 months | 14 (9.9%) | 29 (48.3%) |
| Cases directly transferred from a hospital abroad | 5 (3.5%) | 20 (33.3%) |
| ICU stay within the 12 past months | 49 (34.8%) | 23 (38.3%) |
| **Distribution of cases according to specialty** | | |
| Anesthesia | 6 (4.3%) | 2 (3.3%) |
| Cardiology | 4 (2.8%) | 1 (1.7%) |
| Dermatology | 1 (0.7%) | 2 (3.3%) |
| Gastroenterology | 3 (2.1%) | 8 (13.3%) |
| Gynecology | 1 (0.7%) | 0 (0%) |
| Hematology and Oncology | 11 (7.8%) | 6 (10.0%) |
| Heart and thoracic Surgery | 38 (27.0%) | 6 (10.0%) |
| Infectious Diseases | 1 (0.7%) | 1 (1.7%) |
| Neurosurgery | 2 (1.4%) | 6 (10.0%) |
| Neurology | 2 (1.4%) | 2 (3.3%) |
| Nephrology | 4 (2.8%) | 1 (1.7%) |
| Oral maxillofacial Surgery | 0 (0%) | 1 (1.7%) |
| Pediatric Gastroenterology | 4 (2.8%) | 0 (0%) |
| Pediatric Surgery | 4 (2.8%) | 0 (0%) |
| Pediatric Cardiology | 1 (0.7%) | 1 (1.7%) |
| Pediatric Pulmonology | 5 (3.5%) | 1 (1.7%) |
| Plastic Surgery | 1 (0.7%) | 4 (6.7%) |
| Pulmonology | 1 (0.7%) | 2 (3.3%) |
| Rheumatology | 0 (0%) | 2 (3.3%) |
| Trauma Surgery | 24 (17.0%) | 12 (20.0%) |
| Urology | 13 (9.2%) | 0 (0%) |
| Visceral Surgery | 15 (10.6%) | 2 (3.3%) |
| **Distribution of cases according to positive samples site** (multiple positive body sites possible ) | | |
| Bile | 1 (0.7%) | 0 (0%) |
| Blood | 7 (5.0%) | 3 (5.0%) |
| Nasopharyngeal mucosa | 14 (9.9%) | 20 (33.3%) |
| Rectal | 94 (66.7%) | 27 (45.0%) |
| Respiratory tract secretions | 18 (12.8%) | 17 (28.3%) |
| Skin | 12 (8.5%) | 26 (43.3%) |
| Transplant lung perfusion fluid | 2 (1.4%) | 3 (5.0%) |
| Urine | 23 (16.3%) | 3 (5.0%) |
| Vascular catheter | 3 (2.1%) | 4 (6.7%) |
| Wound/intraoperative | 16 (11.3%) | 21 (35.0%) |
| Other sample site | 3 (2.1%) | 3 (5.0%) |
| **Colonization and infection** (multiple types of infection possible) | | |
| Cases with colonization | 137 (97.2%) | 55 (91.7%) |
| Cases with infection | 24 (17.0%) | 21 (35.0%) |
| Colonization prior or at time of infection | 20 (14.2%) | 15 (25.0%) |
| First sample: colonization | 137 (97.2%) | 54 (90.0%) |
| Bloodstream infection | 7 (5.0%) | 3 (5.0%) |
| Peritonitis | 1 (0.7%) | 1 (1.7%) |
| Pneumonia | 5 (3.5%) | 6 (10.0%) |
| Skin/Soft tissue and surgical site infection | 9 (6.4%) | 13 (21.7%) |
| Urinary tract infection | 6 (4.3%) | 0 (0%) |
| **Cases with co-colonization** | | |
| CR *Escherichia coli* | 10 (7.1%) | 5 (8.3%) |
| CR *Escherichia coli* prior to CR Kp/CR Ab acquisition | 3 (2.1%) | 1 (1.7%) |
| CR *Pseudomonas aeruginosa* | 13 (9.2%) | 7 (11.7%) |
| CR *Pseudomonas aeruginosa* prior to CR Kp/CR Ab acquisition | 2 (1.4%) | 2 (3.3%) |
| CR other GNB | 4 (2.8%) | 6 (10.0%) |
| CR other GNB prior to CR Kp/CR Ab acquisition | 2 (1.4%) | 0 (0%) |
| Methicillin-resistant *Staphylococcus aureus* | 9 (6.4%) | 10 (16.7%) |
| Methicillin-resistant *Staphylococcus aureus* prior to CR Kp/CR Ab acquisition | 7 (5.0%) | 4 (6.7%) |
| Vancomycin-resistant *Enterococcus faecium* | 44 (31.2%) | 18 (30%) |
| Vancomycin-resistant *Enterococcus faecium* prior to CR Kp/CR Ab acquisition | 13 (9.2%) | 8 (13.3%) |

CR = carbapenem resistant, ICU = intensive care unit, IQR = interquartile range, Kp = *Klebsiella pneumoniae*, Ab = *Acinetobacter baumannii*, GNB *=* Gram-negative bacteria

.
