# Supplementary material for: Epidemiology and infection control of carbapenem resistant Acinetobacter baumannii and Klebsiella pneumoniae at a German university hospital: a retrospective study of 5 years (2015–2019)
Source: BMC Infect Dis. 2021 Nov 27;21:1196. doi: 10.1186/s12879-021-06900-3 (PMC8627082; doi:10.1186/s12879-021-06900-3)
Supplement: Supplementary file 2 — Additional file 2. Results of the phenotypic antimicrobial susceptibility testing. [file 12879_2021_6900_MOESM2_ESM.docx]

**Additional file 2.** Results of the phenotypic antimicrobial susceptibility testing

| ***Acinetobacter baumannii*; all isolates: n= 44 (100%)** | | | | |
| --- | --- | --- | --- | --- |
|  | Resistant | Intermediate | Susceptible | Not tested |
| Ciprofloxacin | 40 (90.9%) | - | 4 (9.1%) | - |
| Colistin | 5 (11.4%) | - | 36 (81.8%) | 3 (6.8%) |
| Gentamicin | 32 (72.7%) | - | 12 (27.3%) | - |
| Meropenem | 39 (88.6%) | 5 (11.4%) | - | - |
| Trimethoprim/sulfamethoxazole | 30 (68.2%) | 2 (4.5%) | 12 (27.3%) | - |
| ***Klebsiella pneumoniae*; all isolates: n=79 (100%)** | | | | |
|  | Resistant | Intermediate | Susceptible | Not tested |
| Ceftazidime | 74 (93.7%) | - | 5 (6.3%) | - |
| Ceftazidime/Avibactam | 4 (5.1%) | - | 19 (24.1%) | 56 (70.1%) |
| Ciprofloxacin | 62 (78.5%) | 4 (5.1%) | 13 (16.5%) | - |
| Colistin | 16 (20.3%) | - | 61 (77.2%) | 2 (2.5%) |
| Gentamicin | 40 (50.6%) | 1 (1.3%) | 38 (48.1%) | - |
| Meropenem | 37 (46.8%) | 29 (36.7%) | 13 (16.5%)* | - |
| Piperacillin/Tazobactam | 79 (100%) | - | 0 (0%) | - |
| Trimethoprim/sulfamethoxazole | 54 (68.4%) | 4 (5.1%) | 21 (26.6%) | - |

*despite harboring a carbapenemase, some isolates may present as a susceptible phenotype
