# Supplementary figures and images for: Epidemiology and infection control of carbapenem resistant Acinetobacter baumannii and Klebsiella pneumoniae at a German university hospital: a retrospective study of 5 years (2015–2019)
Source: BMC Infect Dis. 2021 Nov 27;21:1196. doi: 10.1186/s12879-021-06900-3 (PMC8627082; doi:10.1186/s12879-021-06900-3)

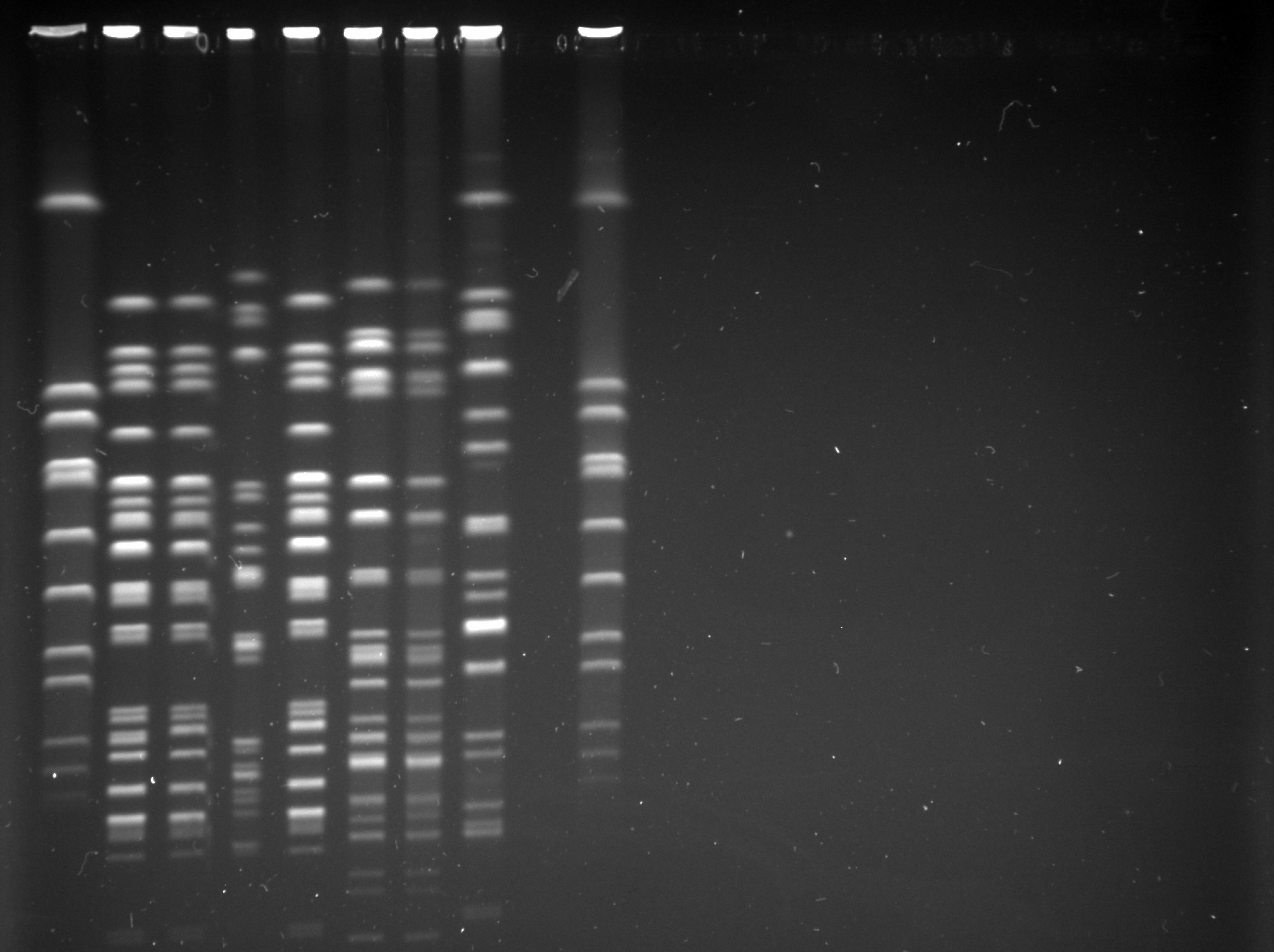

Supplement: Supplementary file 3 — Additional file 3. Pulsed-field gel electrophoresis of the carbapenem resistant Klebsiella pneumoniae cluster in hematology and oncology (original gel). [file 12879_2021_6900_MOESM3_ESM.tif]
